# An Unusual Case of Metastatic Follicular Thyroid Cancer 40 Years after Initial Diagnosis

**DOI:** 10.1155/2018/2019235

**Published:** 2018-12-05

**Authors:** Lakshmi Pakath Menon, Spyridoula Maraka

**Affiliations:** ^1^Division of Endocrinology and Metabolism, Center for Osteoporosis and Metabolic Bone Diseases, University of Arkansas for Medical Sciences and the Central Arkansas Veterans Health Care System, Little Rock, AR 72205, USA; ^2^Knowledge and Evaluation Research Unit in Endocrinology (KER_Endo), Mayo Clinic, 200 First Street SW, Rochester, MN 55905, USA

## Abstract

Thyroid cancer recurrence can occur decades after initial diagnosis despite excellent response to therapy. Thyroid cancer recurrence is evaluated using serum thyroglobulin (Tg) and imaging studies including I-131 WBS and neck ultrasound. Limitations in Tg measurement and WBS may result in failure to detect recurrence. We report the case of a 63-year-old man who was noted to have rhonchi during a routine visit. He had a past history of follicular thyroid cancer that was diagnosed 40 years ago and treated with total thyroidectomy and radioactive iodine. He had excellent response to therapy with undetectable Tg levels, normal neck ultrasounds, and multiple negative whole body scans (WBS) due to which he was discharged from endocrinology clinic after 37 years of follow-up. Chest X-ray revealed a left lung mass with biopsy positive for thyroid cancer. Tg remained undetectable with negative anti-Tg antibody. Left pneumonectomy was done which revealed a mix of 70% differentiated thyroid cancer and 30% poorly differentiated/anaplastic thyroid cancer. He received two cycles of Doxorubicin and Paclitaxel. At 4 months follow-up after surgery, he had 3 subcentimeter nodules in his right lung. This case highlights that physical exam remains an essential tool to evaluate for recurrence. Since the lungs are the most common site of metastasis in follicular thyroid cancer, a chest X-ray may help detect metastasis that is missed on other modalities.

## 1. Introduction

Thyroid cancer accounts for 3.1% of all new cancer diagnosis in the United States and has excellent overall prognosis with 98.1% of all patients surviving at 5 years [1]. Follow-up of thyroid cancer survivors with appropriate surveillance is necessary to monitor for recurrent disease and reduce long term morbidity and mortality. Most cases of thyroid cancer recurrence occur within 10 years of diagnosis but late recurrence may also occur which makes ongoing surveillance important. Recurrence of thyroid cancer may be accompanied by dedifferentiation of the tumor which results in more aggressive behavior [2]. Serum thyroglobulin (Tg) levels, I-131 whole body scans, and neck ultrasounds form the mainstay of surveillance but the limitations of these modalities may result in difficulty in making a timely diagnosis, as highlighted in this case.

## 2. Case Presentation

A 63-year-old man presented to his primary care physician in 2017 for a routine visit and was noted to have left lower lobe rhonchi. He reported that 6 months earlier, he started having wheezing during exercise which improved with time. Review of systems was negative otherwise.

His medical history was significant for follicular thyroid cancer (FTC) treated with total thyroidectomy in 1977 followed by 30 mCi of radioactive iodine (RAI) in 1978. He did not have a history of neck irradiation or a family history of thyroid cancer. He had levothyroxine withdrawal I-131 whole body scan (WBS) in 1984 and 2002 that showed no evidence of residual or metastatic disease. Thyrogen stimulated WBS in 2010 was also unremarkable. He had multiple neck ultrasounds (last in 2014), that did not show any residual thyroid tissue or evidence of local recurrence. Tg levels, including stimulated, were always undetectable with negative anti-thyroglobulin antibody (TgAb). His TSH was maintained less than 2 uIU/ml on levothyroxine 112 mcg daily. The patient was discharged from Endocrinology in 2014 after 37 years of follow-up indicating excellent response to therapy.

**Figure 1:**
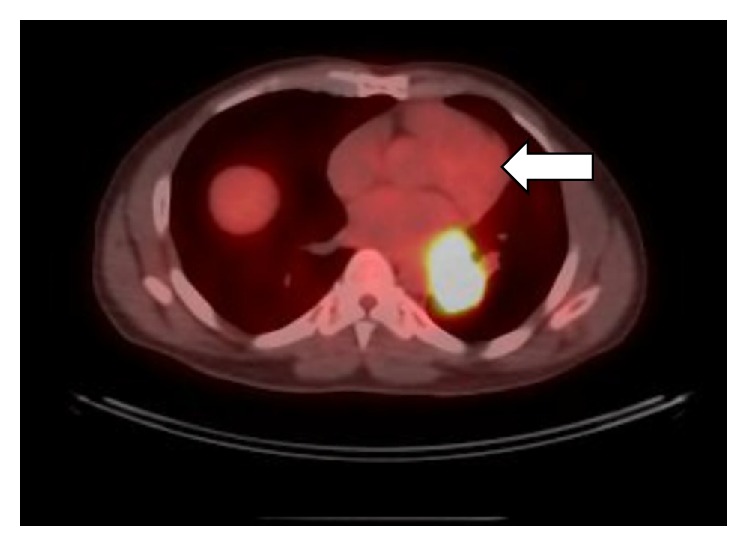
PET CT showing left lobe mass.

**Figure 2:**
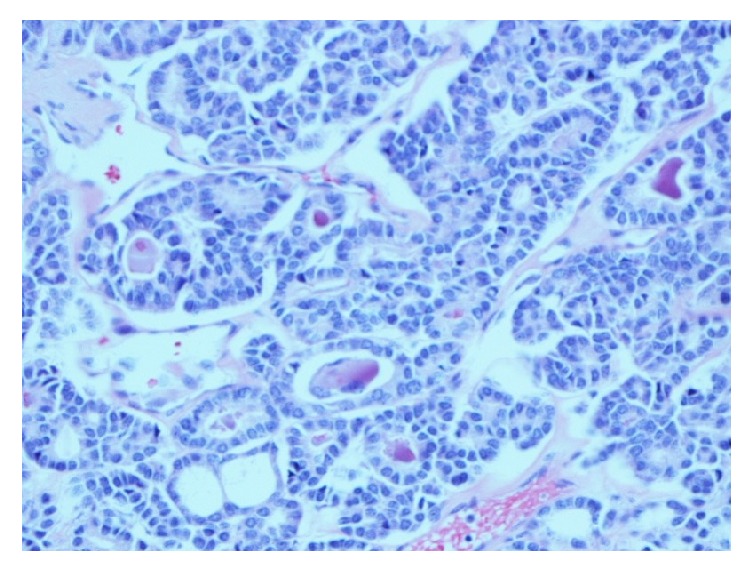
Well differentiated thyroid cancer with colloid.

**Figure 3:**
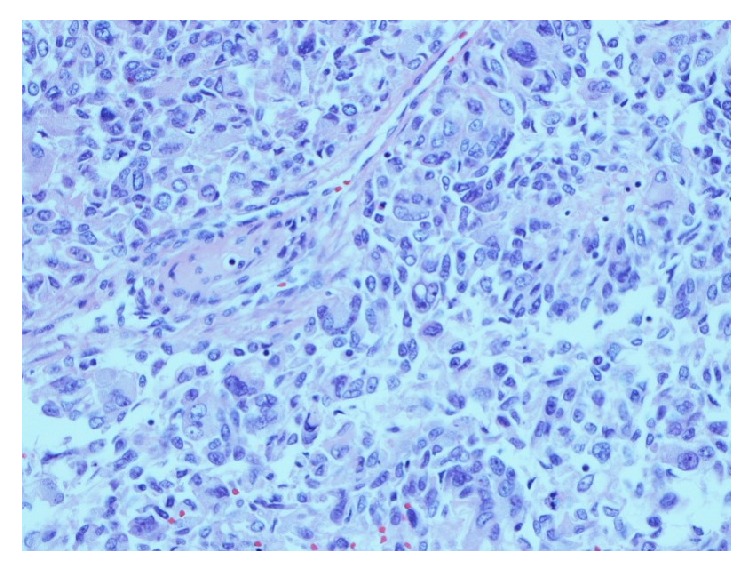
Poorly differentiated thyroid cancer.

A chest X-ray ordered to evaluate the abnormal respiratory finding showed a left paraspinal/periaortic mass. Chest CT showed a 5.3 cm x 3.7 cm x 5.4 cm heterogeneously enhancing left infrahilar mass with occlusion of the left lower lobe bronchus and bilateral pulmonary nodules measuring up to 1 cm on the right and 0.9 cm on the left. He underwent bronchoscopy with endobronchial ultrasound and transbronchial lymph node aspiration. The cytology revealed metastatic FTC. The tumor cells were positive for thyroid transcription factor 1 (TTF-1), cytoketatin-7, focally positive for Tg, negative for cytokeratin-20, synaptophysin, chromogranin, and P63, consistent with thyroid primary malignancy. Thyroid ultrasound showed no evidence of residual thyroid tissue or local cancer recurrence. PET-CT showed a 3.9 cm x 3.7 cm x 5.2 cm FDG avid left lower lobe mass and bilateral indeterminate sub-centimeter non-FDG avid masses (Figure 1). Tg measured by Beckman immunometric assay was undetectable with negative TgAb. Left pneumonectomy and intra-thoracic lymphadenectomy were performed. Final pathology revealed metastatic carcinoma of thyroid with a mixture of well-differentiated FTC (70%) (Figure 2) and anaplastic thyroid carcinoma (ATC) (Figure 3) components. The tumor showed perineural and angiolymphatic invasion. Nine lymph nodes were negative for malignancy. Tumor cells were negative for V600 BRAF mutation. The pathology was reviewed by a second institution which noted that the less differentiated tumor appeared to be poorly differentiated thyroid carcinoma (DTC) rather than ATC. The slides were sent to a national referral center (Defense Health Agency, Silver Spring, MD) which reported that the poorly (DTC) contained within it a small component positive for TTF-1 and negative for Tg, possibly consistent with ATC. Postoperative chest CT, two months following his diagnosis, showed minimal increase in the size of the right pulmonary nodule.

He was treated for presumed ATC with two cycles of Doxorubicin and Docetaxel starting 3 weeks post-operatively. Further chemotherapy was held due to reduction in ejection fraction. RAI could not be used due to repeated administration of iodinated contrast. Patient declined entry in clinical trials. Repeat chest CT 2 months after chemotherapy showed stable lung nodules and a new 4 mm right lung nodule.

## 3. Discussion

FTC is a form of DTC that accounts for up to 12% of cases of thyroid cancer. It has a propensity for hematogenous spread. The most common site of distant metastasis is the lungs followed by the bone [3]. Metastasis has also been reported to skin, brain, liver, adrenal gland, kidney and pancreas [4]. The presence of extra-pulmonary metastasis is associated with worse disease specific survival. Age over 40 and macronodular metastasis (>1 cm) are associated with worse survival [5]. In one study, metastatic disease occurred in around 19% of patients with FTC who were followed for a median of 12 years [6].

Poorly DTC has a behavior that is intermediate between well DTC and ATC. 10 year survival in poorly DTC is around 67% [7]. 5-year survival for ATC is dismal at around 7% with a median survival of only 7 months. Foci of DTC are found in 20-30% of ATC suggesting that ATC may develop as a result of dedifferentiation of pre-existing DTC. RAI is beneficial in poor DTC but has no role in ATC. Histopathologic differentiation between poor DTC and ATC is thus important since it affects prognosis as well as treatment modality. This case highlights the challenges of making a histopathologic differentiation between poorly DTC and ATC. Our patient was initially treated with cytotoxic chemotherapy as a case of ATC since it is the anaplastic component that contributes most to mortality. He had stable imaging 4 months after stopping chemotherapy which subsequently made the diagnosis of ATC less likely.

Most recurrences of thyroid cancer occur within the first decade [8]. Recurrences have been described up to 22 years following thyroidectomy [9]. To our knowledge, this case represents the longest time period after which recurrence occurred and highlights that surveillance for recurrence of thyroid cancer needs to be lifelong, even in patients who are classified as being at low risk of recurrence.

Tg levels are used to evaluate for recurrent or persistent disease in thyroid cancer patients who undergo total thyroidectomy. The American Thyroid Association recommends that the recurrence risk estimates be continuously modified over time based on the patient's clinical course. This patient was classified as low risk due to no structural or biochemical evidence of recurrence on serial evaluations. Negative Tg level may occur if the tumor is poorly differentiated and not capable of producing Tg. In this case, the well-differentiated component stained for Tg despite the negative serum levels. It is possible that the Tg produced by the tumor cells contained structural differences due to which it could not be detected by the immunoassay. Another possibility is that the synthesized Tg was not secreted into the circulation. False negative Tg levels are associated with papillary histology, manifestation in lymph nodes of the neck or mediastinum, and small size none of which was the case here [10].

Iodine 131 WBS is an imaging modality used for the follow up of patients with thyroid cancer. It detects metastasis due to the uptake of I-131 by malignant cells that express sodium iodine symporter (NIS). False negative WBS can occur in poor DTC that lacks expression of NIS. Immunohistochemistry studies show that NIS expression may be absent in around 30% of DTC [11]. Even in malignant cells that express NIS, there may be defects in proper transporting of NIS to plasma membrane which impair its ability to concentrate iodine. False negative rate as high as 40% has been reported with WBS in DTC [12]. It is unclear if our patient had a false negative WBS in 2010 or if the metastasis developed later.

Given the pitfalls associated with utilization of Tg and WBS for follow-up of DTC, additional modalities need to be considered to assess recurrence. FDG-PET can detect cases that are WBS negative but is associated with radiation exposure and high cost if used for routine surveillance. Since the most common site for distant metastasis of FTC is the lungs, a thorough respiratory exam is recommended at each visit. A screening chest X-ray at regular intervals may help detect metastasis that is missed on WBS but the cost effectiveness of this approach is unknown.

This unusual case of FTC highlights the limitations of current modalities for evaluation of recurrence, discusses its possible reasons, and offers a potential approach to overcome them.

## Data Availability

The data used to support this case report is available from the corresponding author upon request.
